# Super-strengthening and stabilizing with carbon nanotube harnessed high density nanotwins in metals by shock loading

**DOI:** 10.1038/srep15405

**Published:** 2015-10-23

**Authors:** Dong Lin, Mojib Saei, Sergey Suslov, Shengyu Jin, Gary J. Cheng

**Affiliations:** 1 School of Industrial Engineering, Purdue University, West Lafayette, IN, 47906, USA; 2 School of Materials Engineering, Purdue University, West Lafayette, IN, 47906, USA; 3 Birck Nanotechnology Center, Purdue University, West Lafayette, IN, 47906, USA; 4 School of Materials and Metallurgy, Wuhan University of Science and Technology, Wuhan 430081, China

## Abstract

CNTs reinforced metal composites has great potential due to their superior properties, such as light weight, high strength, low thermal expansion and high thermal conductivity. The current strengthening mechanisms of CNT/metal composite mainly rely on CNTs’ interaction with dislocations and CNT’s intrinsic high strength. Here we demonstrated that laser shock loading the CNT/metal composite results in high density nanotwins, stacking fault, dislocation around the CNT/metal interface. The composites exhibit enhanced strength with excellent stability. The results are interpreted by both molecular dynamics simulation and experiments. It is found the shock wave interaction with CNTs induces a stress field, much higher than the applied shock pressure, surrounding the CNT/metal interface. As a result, nanotwins were nucleated under a shock pressure much lower than the critical values to generate twins in metals. This hybrid unique nanostructure not only enhances the strength, but also stabilize the strength, as the nanotwin boundaries around the CNTs help pin the dislocation movement.

Carbon nanotubes exhibit super-high strength, stiffness, electrical and thermal properties due to their unique structures^1^,^2^. These superior properties make CNT as ideal reinforcement for metal matrix nanocomposites composites to be used in aerospace and automotive industries^1^,^3^. This strong mechanical properties is due to the exceptional properties of the CNTs, the small mean free path between neighboring CNTs and the great constraint provided by the high surface area of CNTs. Properties of nanomaterials reinforcement are dominated by their surface characteristics, rather than their bulk properties in micronscale reinforcements. The unique interfaces between CNTs and the metal matrix can lead to significant improvements in the mechanical properties. Currently various of methods^1^, have been developed to integrate CNTs into metals, including powder metallurgy, deformation processing, vapor phase processing, solidification processing, electrochemical, laser deposition. In order to further strengthen the composites, high speed torsion and rolling of powder compact CNT/metal composites has been attempted to achieve better mechanical properties^4^,^5^. However, due to the intrinsic low strain rate (less than 10^3^/s) of these methods, the strengthening mechanism is generally dominated by dislocation strengthening and the pining effects of CNTs. In this study, we present a new mechanism to strengthen the CNT/metal interfaces by shock loading.

Dislocation plasticity in nanomaterials reinforced metal composites is controlled by thermal and mechanical activation of sources at the nanomaterials/metal interfaces, a mechanism that requires fluctuations, implying an intrinsic time scale that could explain the reported strain rate sensitivity. This suggests that an increase of the strain rate from 10^4^/s to 10^6^ ~ 10^7^/s, such as in a shock loading, may result in a different regime. During shock loading, lateral relaxation does not have time to occur and pressure builds up. In CNT/metal composites, the pinning effect of CNTs also hinders the escape of dislocations from pile-ups leading to high stresses in front of CNTs. Under these conditions, plasticity is controlled by both high strain rate and high pressure. When the local stresses in front of CNTs exceed the critical stress for twin nucleation, high density deformation twins can be formed.

We present atomistic simulations of shocked CNT/metal composites, in which the extremely short compression time scales are associated with shock loading, and compare the microstructures with those after experimental laser shock loading of CNT/metal composites. The cross section of laser sintered structure is schematically shown in Fig. 1a. multi-wall nanotubes (MWNTs) are integrated into iron matrix by laser sintering (LS)^6^, followed by laser shock peening (LSP) process. Molecular dynamics simulation reveals the high local stress built up around CNT/metal interface, thus enables formations of high density nanotwins. Both MD simulation and experimental results show that nanotwins were nucleated in the iron matrix. The nucleated nanotwins and MWNTs together help greatly increase the strength and stabilize the dislocation movement.

The laser deposition of CNT/Iron composites follows our previous approaches by which both MWNTs^6^ and graphene oxide can be uniformly dispersed and vertically aligned in the cross section of matrix. Due to the large surface area of CNTs, they tends to agglomerate when no dispersants are provided. Here, MWNTs mixed with PVA and layer by layer deposited on substrate^6^. During laser sintering, PVA was vaporized at high temperature when metal and CNTs are molten and solidified^6^. Figure S1 a shows the XRD of various conditions: after coating, after laser sintering and after lasers sintering plus laser shock peening. Iron carbide was generated after laser sintering. We can see that the PVA was fully eliminated after laser sintering from Fig. S1b. The evaporation of PVA bubbles from liquid iron helps aligning MWNTs vertically in the iron matrix^6^. The fast heating and cooling process avoided the aggregation of MWNTs and successfully dispersed MWNTs uniformly in the metal matrix^6^.

Laser shock loading was then performed on the MWNTs reinforced iron composites, as shown in Fig. 1b. A pulse laser transmits the confinement and irradiate the ablative material, forming plasma as the temperature increases dramatically. The expansion of plasma is confined by the confinement layer, resulting in waves propagating in the metal and interacting with metal/MWNTs at high strain rate. The typical microstructures of MWNTs/metal composite after shock loading were demonstrated in Fig. 1d. Figure 1e shows a high resolution TEM image of interfacial area of MWNT and iron matrix. The TEM sample, with certain thickness, was prepared by FIB lift-off method. The observed MWNTs was embedded into the TEM sample. The atomic view of Fig. 1e, which shows highly disordered atoms and rotation, is the overlap view of both MWNT and iron matrix. The circled areas represent the transition of deforming iron atoms under shock loading. The compositions after coating, laser deposition and laser shock loading were measured by XRD, as shown in Fig. S1. In Fig.1f, interior carbon nanotube layer has a diameter of 40.68 Å, armchair (30, 30). Exterior carbon nanotube with a diameter of 81.36 Å is consisted of 120 carbon atoms on equator, armchair (60, 60). The initial structure of each case has been created using MATLAB. Simulation microstructures around MWNTs were captured from (1 1 0) plane represented in Fig. 1g.

Due to experimental constraints, it is difficult to directly measure the dynamic deformation process during high–strain rate loading at the nanoscale. We report large-scale MD simulation on shock loading of MWCNT/Iron composites. Figure 2 shows the atomistic scale stress distribution, disorderness, and defects in the MWCNT/Iron composites during shock loading. The recorded video (movie S1) represents the shock wave propagation in the MWNTs/Iron composite. Figure 2a–c show the atomic pressure of each atom during shock loading, which is calculated in unit of energy. The shock was loaded from bottom and propagated toward the top surface. The maximum pressure at the side of MWNT during the shock loading was 16.075 GPa, which is much higher than the applied shock pressure (10 GPa). This high local stress is built up around MWNTs/metal interface due to presence of dislocation barriers and occurrence of dislocation pile-up. The evolution of centro-symmetry (CS) parameter, disorderness of structure, is shown in Fig. 2d–f. The CS parameter increases after shock wave passing through the CNT/metal composites and leaves the interface with high density of defects. To gain an overall perspective of defects in the structure, we need to distinguish between various defects (dislocations, graphene free surface, stacking faults and twinning). One way to achieve this purpose is to utilize coordinate analysis^7^. Figure 2g–i show the highly disordered and rotated atomic structures at the interfacial area of MWNT and iron matrix under laser shock peening. To describe the defects more clearly, we used common neighbor analysis^8^,^9^ by which it is possible to categorize atoms into BCC, FCC, HCP or unknown atomic structure. Having in mind that seeing FCC and HCP atoms in BCC structure is a defective surface, we know that stacking sequence in HCP and FCC is ABAB and ABCABC, respectively. Therefore we can discriminate stacking faults from other kinds of defects where ever HCP and FCC atoms are beside each other. On the other hand it has been shown^10^ that twining can be perceived as a series of stacking faults following each other, therefore an interlayer of FCC and HCP in iron matrix is indicator of twining. We can locate dislocations and free surface by unknown atomic structure (grey). Also Dislocation Extraction Algorithm has been used to obtain 1D and 2D defects inside the structure after shock process. Figure 2j represent result achieved from DXA code^11^. It shows high density of stacking fault and twin boundaries around the MWNTs.

The shock wave passing through the beginning (Fig. 3a–c) and end (Fig. 3d–f) of MWNT were also investigated. In Fig. 3a,d, we can see low pressure distribution at beginning and end of MWNTs. In corresponding to this phenomenon, lower CS values were also observed at beginning and end of MWNTs. It is originating from the fact that on both ends of MWNT the shock wave interacts with open space within rings of carbon nanotube instead of lateral walls of CNTs. It makes a very small dead metal zone in response to the shock propagation. It also demonstrates how MWNT works as impede for shock propagation and will reflect the shock away. This reflection can be another source of highly defective structure surrounding CNTs.

The flow stress during shock process has been monitored in MD simulation for both pure iron and Fe/CNT composite after 4000 time steps. As shown in Fig. 3g,h the shock front is on 280 Å point for both iron matrix and CNT composite. The flow stress behind the shock front is 9e6 bars.Å^3^ and 12.5 e6 bars.Å^3^ for pure iron and CNT composite, respectively. It shows a 39% increase in strength for CNT composite. There is a decrease in the flow stress behind the shock in CNT case that can be the result of interactions of shock with carbon atoms. It is interesting to see that peak for shock front is weaker for CNT composite. It displays how CNT plays a critical role in the way shock propagates into iron matrix. The roughness of flow stress curve in CNT composite is another support for strong interactions of iron-carbon during shock process.

In addition to the simulations shown above, we have performed experiments to understand the behavior of CNT/metals composites under extreme conditions. The experimental results are in agreement with our atomistic simulations. Although the exact dislocation density in recovered samples is difficult to estimate, our high-resolution TEM images do show residual dislocations inside some nanograins (Fig. 4). This is quite unusual in nanocrystalline materials and not easily achievable under normal deformation conditions^12^. After shock loading, a more focused and interested view of microstructures can be seen in Fig. 4a,b, which is viewed along <110> zone axis. The microstructure evolution process is shown in movie S2. It demonstrates the generation and propagation of nanotwins and other microstructures. In Fig. 4a multiple twin boundaries is shown and Fig. 4b shows coherent single twin boundary. In Fig. 4a,b all non-bcc atoms have been shown in white, while bcc atoms are presented in blue. In Fig. 2g–i only atoms which belong to no specific atomic structure are colored as white. The cross-sectional microstructure was carefully characterized by high resolution TEM and twined structure was discovered. Figure 4c shows multi-twin structure with non-coherent boundaries around a carbon nanotube. The close-up view of one coherent nanotwin is shown in high-magnification TEM figure in Fig. 4d, which is a 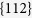 twin. The coherent twin boundary (TB) is marked in Fig. 4d. The insert image is its selected area diffraction pattern (SAED). The insert diffraction pattern further proves the nanotwin structure in Fig. 4d. The results in Fig. 4 show that the simulation and experimental results match each other. MD simulation provides a powerful tool to reveal the progress of microstructure evolution under shock loading.

The generation of twins in bcc iron by laser shock loading was reported at a high pressure of 13.2 GPa^13^,^14^. Large scale of microtwins was observed in shock-loaded (~16.4 GPa) iron^15^. The measured maximum pressure of shock peened iron matrix around MWNT is 16.075 GPa. This value is much higher than the critical values for nucleating twins in bcc iron. Twins in metals and alloys beyond critical pressure can be induced by planar shock loading^7^. A twin initiates from a region of planar slip and it thickens with increasing plastic strain^16^. The similar crystallographic and morphological features generated by conventional and shock deformed twins lead to the conclusion that the mechanisms responsible for the nucleation and growth should be similar^17^. Twining and slip should always begin at the most highly stressed system^16^. In our paper, this area around the side of MWNT has the highest stress concentration. It also explains why we can observe nanotwins in Fig. 4a,b from this area. Some other results also revealed that additional twining was discovered at the adjoining region of second phase particles and matrix by two factors: interface stress and shock wave reflected, refracted, and disturbed by passing through particles^17^. The inclusion has a clear effect on the distribution of deformation twins. The region “down stream” of inclusion has a higher hardness than average, while the residual hardness is related to the square root of the peak pressure^7^. Stress was shown to be concentrated in and around the nanoparticles after LSP^18^. Thin twins would be formed at lower shock induced pressures since dislocation emission and the activation of glide processes are pressure dependent^17^.

High density of dislocations was discovered in the cross-section of TiN nanoparticles reinforced metal composite after LSP^19^. However there is no nanotwin generation since the stress localization caused by shock wave interaction with nanoparticles^20^ is not high enough for emitting nanotwins. Our MD simulation also shows that the stress is concentrated around the CNT. The localized stress results in the nucleation of nanotwins near the side of MWNTs after shock loading. The abruptly stop of dislocation movement at high strain rate deformation can emit a twin plate which immediately takes over as the dominant mode of plastic deformation^21^. High concentration of strain energy is required for the formation of twinning and this can be provided by the stress concentration. Because concentrated stress decreases fast away from MWNTs, the dislocations generated by shock loading may not be able to move far from the sources. The motion of the dislocations is inhibited by nano-sized carbon nanotubes, leading to bending of dislocations^22^. This phenomenon is so called Orowan looping. It produces a back stress, prevents further dislocation migration and results in local emitting of nanotwins around MWNTs^23^. Apparently, this localized stress concentration cannot affect the overall response of the materials under applied shock loading^23^. However, with higher weight ratio and well distributed carbon nanotubes, it is possible to generate highly dense nanotwins in Fe/CNT nanocomposites.

Iron matrix strengthening effect under different surface processing conditions is shown in Fig. 5. The surface micro-hardness (Vickers hardness) (Fig. 5a) of as-received sample is 310 VHN. The surface hardness after laser sintering 11 wt. % of TiN nanoparticles increased to 410 VHN^19^,^24^,^25^, while the hardness increased to 605 VHN when 2 wt. % of MWNTs was integrated into iron matrix. Surface hardness is related to dislocation density: 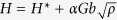, where H^*^ and *α* are materials’ constants, *G* is the shear modulus, *b* is Burger’s vector, and 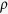 is dislocation density. Dislocation density increases by thermal expansion mismatch can be expressed by^22^:


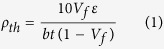


where *V*_*f*_ is the volume fraction of MWNTs, *ε* is the thermal strain, *b* is the Burgers vectors, and *t* is the diameter of the MWNTs. As the peak pressure, *P* (in plane-wave compressive shock loading), increase, both dislocation density and surface hardness were increased. The surface hardness of LS plus LSP of 11 wt. % of TiN nanoparticles^19^,^25^ and 2 wt. % of MWNTs are 550 and 645 VHN, respectively. The hardness increased by 108% comparing the base material with the compoisite after LS plus LSP by integrating 2 wt. % MWNTs.

In order to measure the stress strain relationship of these thin layers, instrumented indentation using a spherical tip was carried out to provide estimates of the stress strain behavior of the samples under three processing conditions: (1) after laser shock peening of laser sintered CNT/Fe composite; (2) after laser sintering of CNT/Fe composite; (3) as received sample. As shown in Fig. 5b, the typical stress strain curves for the three conditions, the yield strength of sample after LSP plus LS (is about 100% higher than that of as received sample, and about 50% higher than that of the sample after LS. The testing methos and interpration of the stress strain can be found in supporting online materials.

During LSP, dislocations can be piled up by MWNTs and also generated nanotwins and dislocation density would be increased during plastic deformation^10^.


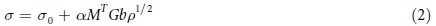


where *σ* is the flow stress, *σ*_0_ is the friction stress, *α* is the constant (1/3), *M*^*T*^ is Taylor factor (3 for untextured polycrystalline materials)^26^, *b* is the burgers vectors, and *ρ* is the dislocation density. Dislocation movement is inhibited by carbon nanotube and nanotwins. The back stress, as results of shock wave interact with CNTs, will increase the resistance for further movement of dislocations and also increases the yield stress^22^. Deformation twins can also strengthen the materials due to a reduction of effective slip length (Hall-Petch effect) and an increase of hardness in the twinning area (Basinski Mechanism)^22^. These effects together contribute the increase of strength.

The thermal stability of surface work hardening was also investigated by annealing under 350 °C. In the previous study, it was found that TiO_2_ nanoparticles not only harden the materials, but also lock up dislocations by pinning effects^7^. The nanoparticles can also prevent dislocations from annealing out^7^. Our previous study also shows that integrated nanoparticles helped increase the thermal stability of surface hardness^19^. Figure 6a schematically shows the dislocation pile-up by multiple twins around MWNTs. Several layers of nanotwin planes blocked the dislocation movement in the iron matrix. Figure 6b shows that the surface hardness of LS of 2 wt. % MWNTs dropped from 550 VHN to 430 VHN after 20 minutes of annealing, which decreased around 22%. The micro-hardness finally reached 320 VHN after 5 minutes. On the other hand, the surface micro-hardness of the sample after shock loading decreased to 630 VHN after 200 minutes of annealing and it slowly dropped to 625 VHN after 500 minutes. The hardness only decreases by 3% after 500 minutes of annealing. Two significant microstructures exists in the cross section—MWNTs and nanotwins. MWNTs blocked the dislocation movement by Orowan looping effect^22^. Twin boundaries are viewed as line in the TEM figures, however they are actually 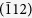 twin planes in the cross section, which are parallel to the direction we obtained TEM figures. These twin boundaries were also served as barriers for the dislocation movement and greatly stabilized the dislocation movement. Basinski *et al.* proposed that glissile dislocations before twining were converted to sessile dislocation after the generation of twins^12^,^22^, which improves the thermal stability of dislocations.

In summary, the interaction of CNTs and laser shock wave has been studied by experiment and simulation to explore the beneficial nanotwin structures for better mechanical properties. This report presents a technique to control the density and distribution of nanotwins by designing the concentration and distribution of CNTs in metals and realizing engineered nanotwin structure in bulk metallic structures. It is found that the unique hybrid nanostructures results in enhanced strength with excellent stability. The results in this report open new ways to fabricate super-strong and stable structures with wide variety of applications. Extremely high stress localization are developed surrounding the CNT during the laser shock loading, much higher than the applied shock pressure, which helps emit high density nanotwins. As a result, nanotwins were nucleated under a shock pressure much lower than the critical values to generate twins. This work open a direction to make use of high strain rate to generate nanostructures in carbon based nanocomposites.

## Methods

### Materials

AISI 4140 was selected as the substrate. The heat treatment process can be found in our former studies^6^,^19^,^27^. After thermal treatment, surface micro-hardness was measure as 310 VHN. The iron powders (average diameter of 4 μm) and multi-wall carbon nanotubes (from Cheaptube Inc.) were used for the laser sintering. The outside and inside diameters of MWNTs are 8–15 nm and 3–5 nm, respectively. The length of MWNTs is 10–50 μm.

### Laser sintering

Micro-sized iron (1.96 g) powder and MWNTs (0.04) were mixed in 46 g DI water. Polyvinyl alcohol (PVA), around 2 g, was added in the suspension in order to separate MWNTs^6^,^19^,^28^. The suspension was prepared by stirring on hotplate at 90 °C for more than 12 hours. The suspension was coated on the substrate surface, which had been mechanically polished, and dried in lab circumstance^29^. During laser sintering, IPG fiber laser was working at 50 KHz and 100 W. The N_2_ gas was filled the chamber in order to avoid oxidation through the whole process.

### Laser shock peening

LSP was performed after LS. A Nd:YAG laser system (wavelength 1064 nm and pulse length 5 ns) was used for LSP. The details of LSP process can be found in our previous paper^19^. LSP was then performed on the nanocomposites with laser intensity of 4 GW/cm^2^ and the calculated peak pressure is 8.662 ± 1.614 GPa^19^.

### Microstructure characterization

The cross sectional microstructure features were characterized by the FEI Titan system operating at 300 keV and the TEM samples were prepared by lift-out method by using FEI Nova 200 focused ion beam (FIB). The composition was characterized by Bruker D8 focus X-Ray diffractiometer using Cu-K_α_ source.

### MD simulation

In this work LAMMPS package^30^ was used for MD simulation of shock propagation through iron MWNT composite. Width of simulation box is set to 30 nm to count for repetition of CNT in distances of 30 nm to maintain the experimental weight ratio of composite. MD simulation has been done in two stages: First, whole structure has been implemented unrestricted with periodic boundary condition in all directions to reach equilibrium condition. Second, impactor of iron atoms with speed of 1.2 km/s which is equivalent with 9.72 GPa pressure is induced.

### Mechanical property testing

The micro-hardness of initial AISI 4140, sample with LS and sample with LS plus LSP was measured by Leco M-400-H micro-hardness instrument with 300 g load and 10 s holding time.

### Stress strain curves

Instrumented indentation using a spherical tip was carried out to provide estimates of the stress strain behavior of the samples. Experiments were performed in a Hysitron Triboindenter 950, using a nominally spherical diamond tip with a tip radius of 4970 nm, as calibrated by performing elastic indentations into tungsten single crystals and indentations into fused quartz. The load—partial unload method developed by Field and Swain^31^ was utilized to make 10 unique indentations in each material.

## Additional Information

**How to cite this article**: Lin, D. *et al.* Super-strengthening and stabilizing with carbon nanotube harnessed high density nanotwins in metals by shock loading. *Sci. Rep.* **5**, 15405; doi: 10.1038/srep15405 (2015).

## Supplementary Material

Supplementary Information

Supplementary Movie S1

Supplementary Movie S2

## Figures and Tables

**Figure 1:**
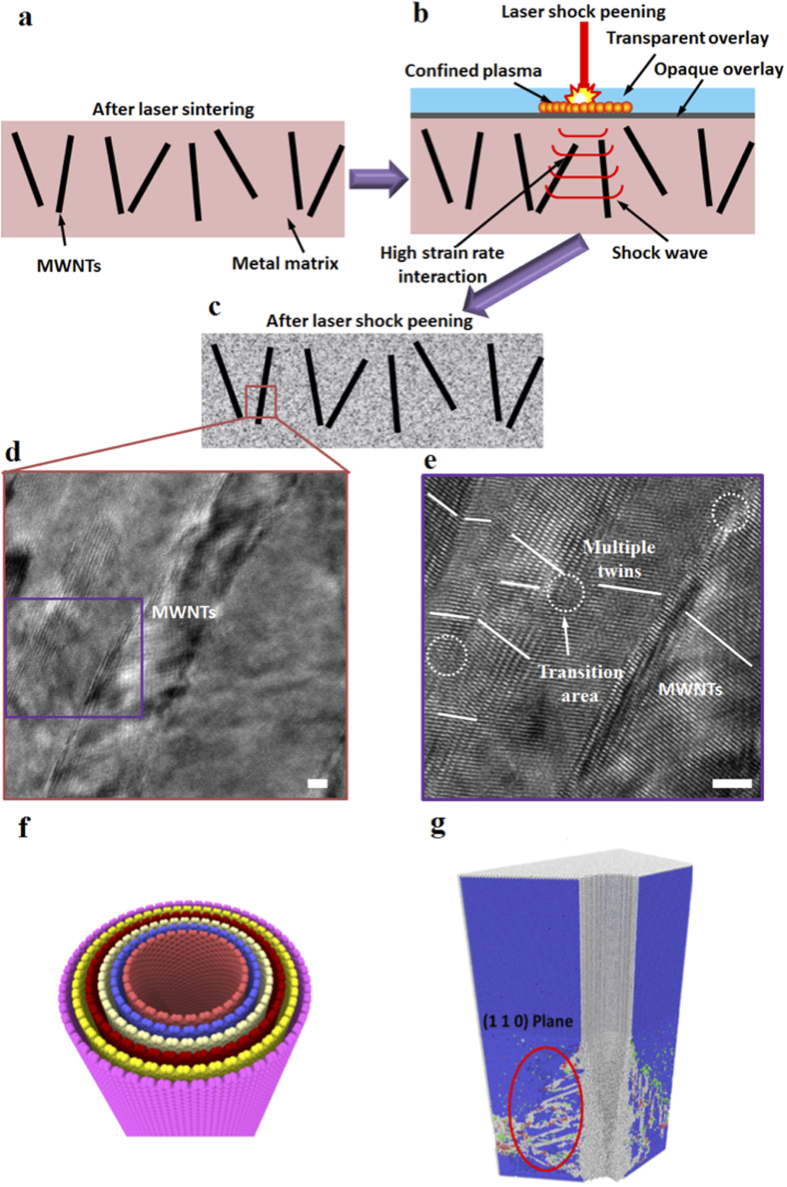
Overview of laser shock wave interaction with CNT/metal composites. (**a**) Cross sectional view of MWNTs in iron matrix after LS. (**b**) Schematic of LSP process, shock wave interacting with metal and MWNTs generating high strain rate deformation. (**c**) Cross section of Fe/MWNTs after LSP. (**d**) TEM image of MWNTs after LSP, scale bar: 2 nm. (**e**) High resolution TEM image of selected area in (**d**) showing interface microstructure around MWNT, scale bar: 2 nm. (**f**) Structure of MWCNT inside iron matrix. (**g**) Cross sectional view after MD simulation showing the twin nucleation around CNTs in bcc structure.

**Figure 2:**
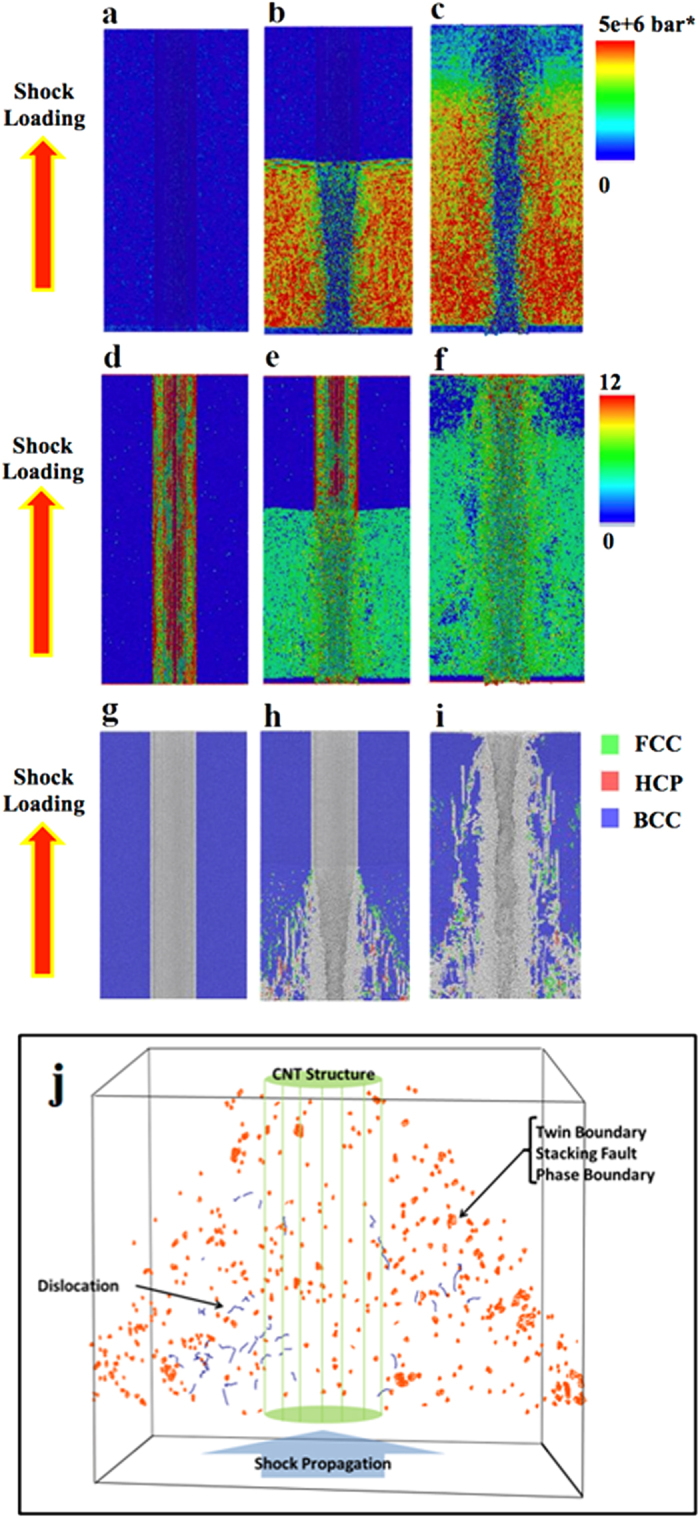
Snap shots of shock loading of MWNT/Iron composite by MD simulation. (**a–c**) Pressure distribution. (**d–f**) Disorderness represented by centro-symmetry parameter. (**g–i**) Defects distribution during shock loading. (**j**) dislocations and twin boundaries inside the shocked structure.

**Figure 3:**
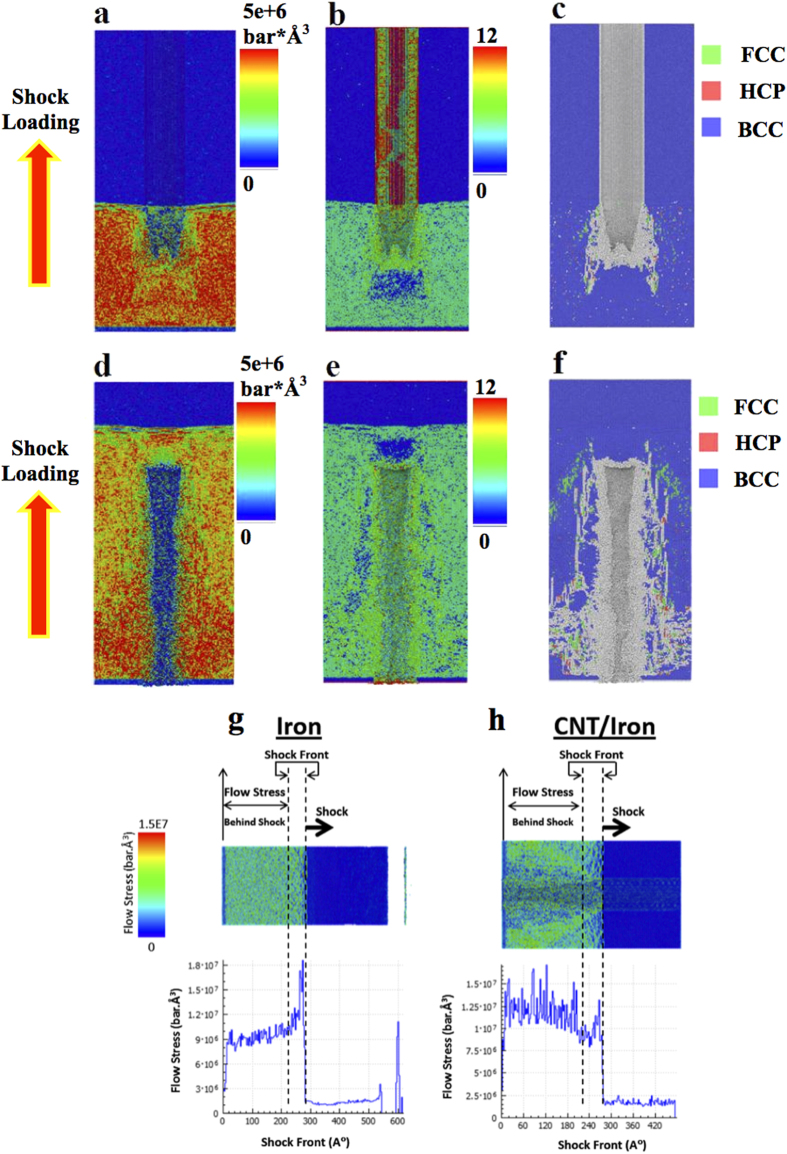
MD simulation on shock front passing through the beginning and end of MWNT. Pressure distribution at the (**a**) beginning and (**d**) end of CNT. Centro-symmetry parameter at the (**b**) beginning and (**e**) end of CNT. Shock loading generated defects at the (**c**) beginning and (**f**) end of CNT. (**g**) flow stress as the shock wave passes the iron and **(h)** CNT/iron cells respectively.

**Figure 4:**
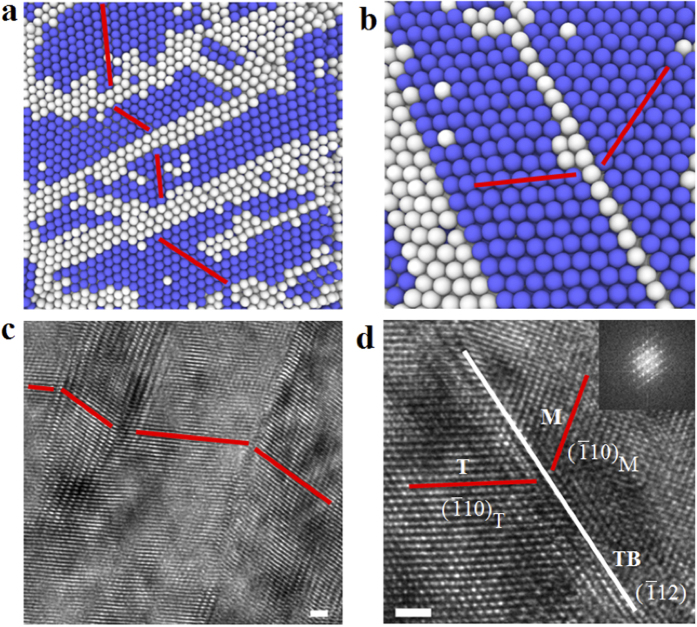
Atomic view of nanotwins. (**a**) Molecular dynamic simulation results of multiple twins with non-coherent twin boundaries generated after shock loading. (**b**) Nanotwin with coherent boundary. (**c**) High resolution TEM image of multiple twins with non-coherent twin boundaries. (**d**) High resolution TEM image of nanotwin with coherent boundary. Scale bar in (**c,d**): 1 nm.

**Figure 5:**
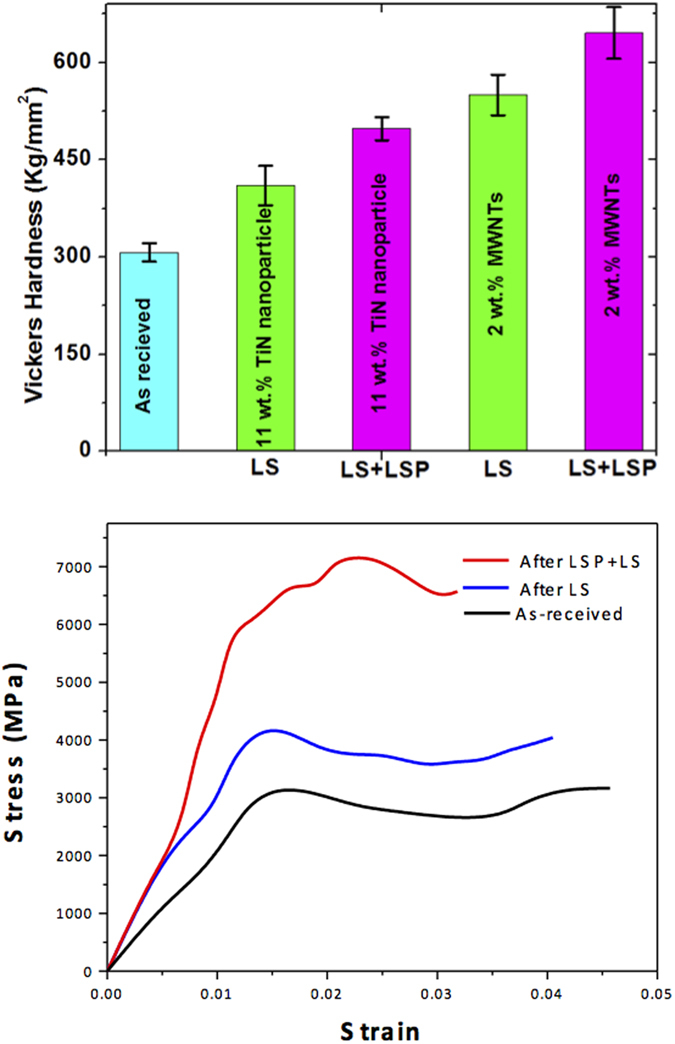
Strength testing results. (**a**) Surface Micro-hardness of samples after various processing conditions, including as received, LS of 11 wt. % TiN nanoparticles, LS plus LSP of 11 wt. % TiN nanoparticles, LS of 2 wt. % of MWNTs, and LS plus LSP of 2 wt. % MWNTs. (**b**) Stress strain curves of samples after various processing conditions, including as received, LS of 2 wt. % of MWNTs, and LS plus LSP of 2 wt. % MWNTs.

**Figure 6:**
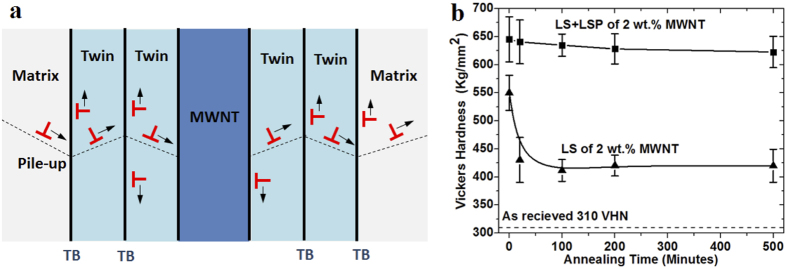
Ultra-high stability of dislocation. (**a**) Schematic illustration of dislocation pile-up along multiple twins around MWNT. (**b**) Thermal stability of surface micro-hardness of LS of 2 wt. % MWNTs and LS plus LSP of 2 wt. % MWNTs.

## References

[b1] Arvind Agarwal S. R. B. & Debrupa Lahiri. Carbon Nanotubes Reinforced Metal Matrix Composites. CRC Press (2011).

[b2] Li H., Zhang Q. & Xiao H. Self-deicing road system with a CNFP high-efficiency thermal source and MWCNT/cement-based high-thermal conductive composites. Cold Regions Science and Technology 86, 22–35 (2013).

[b3] Hwang J. Y., Neira A., Scharf T. W., Tiley J. & Banerjee R. Laser-deposited carbon nanotube reinforced nickel matrix composites. Scripta Mater 59, 487–490 (2008).

[b4] Yoo S. J., Han S. H. & Kim W. J. A combination of ball milling and high-ratio differential speed rolling for synthesizing carbon nanotube/copper composites. Carbon 61, 487–500 (2013).

[b5] Jenei P., Gubicza J., Yoon E. Y., Kim H. S. & Lábár J. L. High temperature thermal stability of pure copper and copper–carbon nanotube composites consolidated by High Pressure Torsion. Composites Part A: Applied Science and Manufacturing 51, 71–79 (2013).

[b6] Lin D., Richard Liu C. & Cheng G. J. Laser sintering of separated and uniformly distributed multiwall carbon nanotubes integrated iron nanocomposites. Journal of Applied Physics 115, 113513 (2014).

[b7] Blazynski T. Z. Materials at high strain rates. Elsevier applied science (1987).

[b8] Faken D. & Jónsson H. Systematic analysis of local atomic structure combined with 3D computer graphics. Comp Mater Sci 2, 279–286 (1994).

[b9] Tsuzuki H., Branicio P. S. & Rino J. P. Structural characterization of deformed crystals by analysis of common atomic neighborhood. Computer Physics Communications 177, 518–523 (2007).

[b10] Lahiri D., Bakshi S. R., Keshri A. K., Liu Y. & Agarwal A. Dual strengthening mechanisms induced by carbon nanotubes in roll bonded aluminum composites. Materials Science and Engineering: A 523, 263–270 (2009).

[b11] Alexander S. & Karsten A. Extracting dislocations and non-dislocation crystal defects from atomistic simulation data. Modelling and Simulation in Materials Science and Engineering 18, 085001 (2010).

[b12] Mahajan S. & Williams D. F. Deformation Twinning in Metals and Alloys. International Metallurgical Reviews 18, 43–61 (1973).

[b13] Barker L. M. & Hollenbach R. E. Shock wave study of the α to ε phase transition in iron. Journal of Applied Physics 45, 4872–4887 (1974).

[b14] Rittel D., Ravichandran G. & Venkert A. The mechanical response of pure iron at high strain rates under dominant shear. Materials Science and Engineering: A 432, 191–201 (2006).

[b15] Wang S. J. *et al.* “Microstructural fingerprints of phase transitions in shock-loaded iron.” Scientific Reports 3 (2013). doi: 10.1038/srep01086 PMC3548189 23336068

[b16] Mahajan S. Interrelationship between slip and twinning in BCC crystals. Acta Metallurgica 23, 671–684 (1975).

[b17] Meyers M. A. & Murr L. E. Shock-waves in high-strain-rate phenomena in metals. Plenum Press: New York, (1981).

[b18] Mahajan S. Effects of existing substructure on shock-twinning behaviour of iron. physica status solidi (a) 2, 217–223 (1970).

[b19] Lin D. *et al.* Mechanism of fatigue performance enhancement in a laser sintered superhard nanoparticles reinforced nanocomposite followed by laser shock peening. Journal of Applied Physics 113, 133509 (2013).

[b20] Ye C. & Cheng G. J. Laser shock peening of nanoparticles integrated alloys: numerical simulation and experiments. Journal of Manufacturing Science and Engineering 132, 061017 (2010).

[b21] Friedel J. Dislocations. Pergamon Press, Oxford (1964).

[b22] George R., Kashyap K. T., Rahul R. & Yamdagni S. Strengthening in carbon nanotube/aluminium (CNT/Al) composites. Scripta Mater 53, 1159–1163 (2005).

[b23] Priestner R. & Leslie W. C. Nucleation of deformation twins at slip plane intersections in B.C.C. metals. Philosophical Magazine 11, 895–916 (1965).

[b24] Lin D. A laser sintered layer of metal matrix consisting of 0D, 1D and 2D nanomaterials and its mechanical behaviors. In: Industrial Engineering (ed^(eds). PURDUE UNIVERSITY (2013).

[b25] Lin D. *et al.* Mechanism of Fatigue Performance Enhancement in a Superhard Nanoparticles Integrated Nanocomposites by a Hybrid Manufacturing Technique. In: ASME 2013 International Manufacturing Science and Engineering Conference collocated with the 41st North American Manufacturing Research Conference (ed^(eds). American Society of Mechanical Engineers (2013).

[b26] Yu S., Liu Y., Ren L. & Li W. Development of laser-cladding layers containing nano-Al_2_O_3_ particles for wear-resistance materials. Metallurgical and Materials Transactions A 37, 3639–3645 (2006).

[b27] Lin D. *et al.* Laser direct writing of crystalline Fe2O3 atomic sheets on steel surface in aqueous medium. Applied Surface Science 351, 148–154 (2015).

[b28] Lin D., Richard Liu C. & Cheng G. J. Single-layer graphene oxide reinforced metal matrix composites by laser sintering: Microstructure and mechanical property enhancement. Acta Mater 80, 183–193 (2014).

[b29] Lin D. *et al.* Laser assisted embedding of nanoparticles into metallic materials. Applied Surface Science 258, 2289–2296 (2012).

[b30] Plimpton S. Fast Parallel Algorithms for Short-Range Molecular Dynamics. Journal of Computational Physics 117, 1–19 (1995).

[b31] Field J. S. & Swain S. V. A simple predictive model for spherical indentation, J. Mater. Res. 8(2), 297–306 (1993).

