# Supplementary material for: Super-strengthening and stabilizing with carbon nanotube harnessed high density nanotwins in metals by shock loading
Source: Sci Rep. 2015 Oct 23;5:15405. doi: 10.1038/srep15405 (PMC4615980; doi:10.1038/srep15405)
Supplement: Supplementary Information [file srep15405-s1.doc]

**Super-strenghening and stabilizing with carbon nanotube harnessed high density nanotwins in metals by shock loading**

Dong Lin^1,3^, Mojib Saei^1,3^, Sergey Suslov^2,3^ , Shengyu Jin^1,3^, Gary J. Cheng^1,3*^

Movie S1

Movie S2


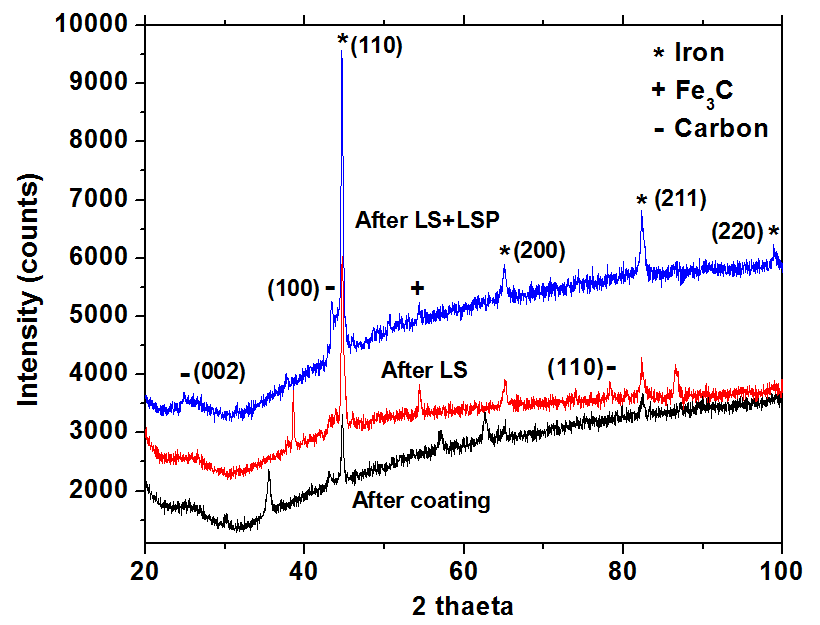


(a)


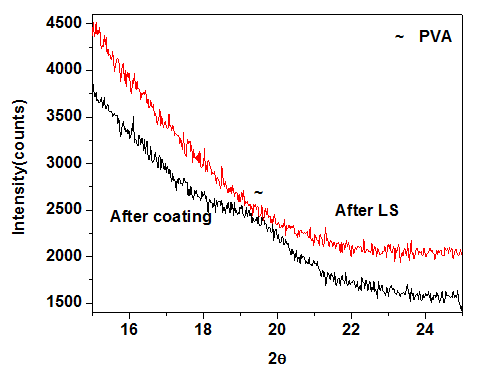


**Supplementary Figure S1 │ XRD curves (a) after coating, after LS and after LS plus LSP and (b) after coating and after LS showing PVA eliminated after laser sintering.**

**Molecular dynamic simulation**

Two materials are included in each simulation setup Fig S2.a: matrix made of iron with bcc lattice structure and lattice constant of 2.87 Ȧ while [1 0 0] lattice vector aligned in x direction Fig S2.b, multi-wall carbon nanotube consisted of 6 layers of single-wall carbon nanotube. Structures shown in Fig 2 and 3 of manuscript are taken from cut shown in Fig S2.c. This is a cut of the middle of structure while CNT has been deleted to get an unimpeded view from matrix. Simulation microstructures shown in Fig 4.a and b of main script is captured from (1 1 0) plane represented in Fig S1.d.

We run 3 different configurations to examine interactions of iron background with carbon nanotube in middle part, head, and tail, respectively. First structure is Multi-Wall Carbon NanoTube (MWNT) inside iron matrix, with both sides of CNT reaching the boundary of simulation. Second configuration is consisted of first structure plus an additional iron matrix below CNT (shock will pass additional iron matrix before CNT). Third structure is just like latter, but added iron matrix is on top of CNT. Additional iron matrix is 18 nm thick, which considering the thickness of main part from first structure (53 nm) make a free path of 71 nm for shock to pass though.

The adaptive intermolecular reactive empirical bond order (AIREBO)[^1^](#_ENREF_1) potential is used for interactions of carbon atoms inside each single-wall carbon nanotube. To avoid spuriously long atomic interactions of carbon atoms 2.0 Ȧ is chosen as cutoff distance. EAM potential[^2^](#_ENREF_2) extracted from Mendelev *et al.* work[^3^](#_ENREF_3) has been used for iron. The interactions of carbon-iron, and carbon-carbon for interlayer carbon nanotube atoms has been defined using standard 12/6 Lennard-Jones potential. Table 1 shows LJ parameters used in this work. Carbon-iron interaction parameters have been extracted using Lorenz-Berthelot[^4^](#_ENREF_4) mixing rule.

**Table 1│** **Lennard-Jones C-Fe and C-C parameters** .

| Interaction | $\varepsilon$ (eV) | $\sigma$ (Ȧ) |
| --- | --- | --- |
| C-Fe | 0.05 | 3.1 |
| C-C | 0.0035 | 3.5 |

Shock is produced using a plane of iron atoms impacting the structure. Impactor is consisted of 4 unit cell of iron atoms. Periodic boundary condition is implemented in planar directions. While in shock direction free boundary condition is established.

Starting with a random initial velocity distribution equivalent with 300 K, Isothermal–isobaric ensemble ensemble (NPT) was implemented for 1 ps with a time step of 0.5 fs to reach equilibrium conﬁguration. During equilibrium step periodic boundary condition is implemented in all 3 directions. Before the beginning of shock process, free boundary condition will be established in shock direction. Then the impactor with speed of 1.2 km/s will strike the structure using microcanonical ensemble (NVE). To obtain visualization of atoms and properties in atomic scale Ovito package has been used[^7^](#_ENREF_7).

The realation between shock speed and shock pressure can be obtained from Fabbro *et al.* [^8^](#_ENREF_8):

$V=(2\mathrm{P}\xi)/\mathrm{m}$ (1)

where $\xi$, $\mathrm{P}$, and $V$are laser pulse duration, pressure and velocity of shock wave, respectively. While m is per area weight of ablation layer defined by:

$\mathrm{m}=\rho\mathrm{e}$ (2)

Where $\rho$and $\mathrm{e}$are density and thickness of ablation layer, respectively. Considering experimental shock pressure as 8.662$\pm$1.614 GPa, we expect shock velocity to be in range of 0.87 to 1.27 km/s.


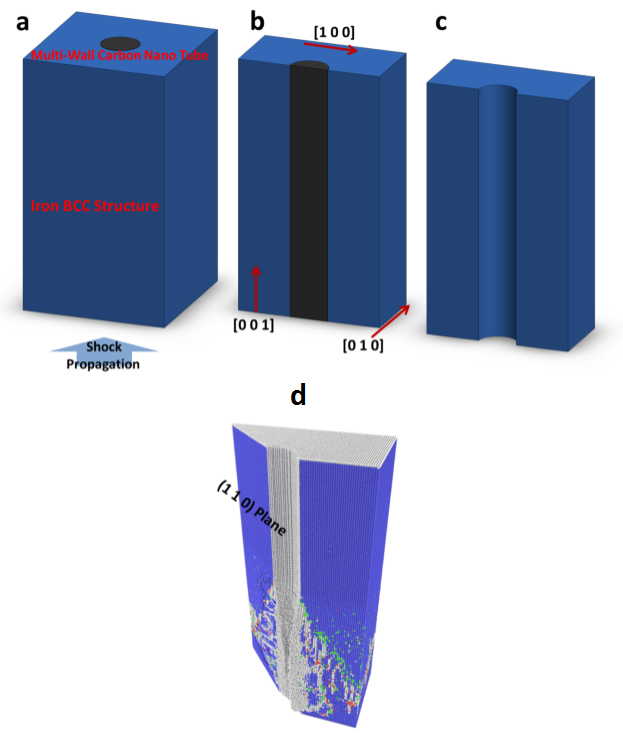


**Supplementary Figure S2 │** (a) Overall structure of simulation box (b) specific atomic directions corresponding to primary direction of 3D structure (c) cut used for showing the results in this paper (d) plane used for finding twins in iron structure and area of twin nucleation in bcc structure.

**Measurement of stress strain curves:**

Experiments were performed in a Hysitron Triboindenter 950, using a nominally spherical diamond tip with a tip radius of 4970 nm, as calibrated by performing elastic indentations into tungsten single crystals and indentations into fused quartz. The load - partial unload method developed by Field and Swain was utilized to make 10 unique indentations in each material, with 15 load-unload segments for each indentation with total time of 45 sec. The maximum load applied by the transducer in any indentation was 13 mN, and the segment maximums were selected to evenly select loads between 50 μN and the transducer maximum (i.e. Unloading was carried out at 50, 200, 500, 900, 1400 μN, etc). The unloading segment was to approximately 50% of the previous maximum load of any given segment. The loading time between segments was 1 second, with a 1 second hold, and then 1 second unload. The contact depth, segment maximum load, and unloading stiffness was determined for each segment of each indentation, and then the method of Field and Swain was applied to calculate the nominal indentation stress-strain behavior. Using Field and Swain method ^9,10^, the representative stress and strain can be calculated based on the following formulas:


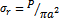
 (3)


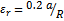
 (4)

where *P* is the maximum load of cycle, *R* is the radius of indenter and *a* is the radius of the circle of contact which can be calculated from:


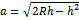
 (5)

Considering *h* as the contact depth. Care should be taken in interpreting of the strain-stress curve to not compare it with compression test, as this methodology is based on assumption of partial unloading to be purely elastic and Hertz’s theory is utilized to estimate the contact radius. Furthermore it should be note that the estimate of the contact radius based on Hertz’s theory emanates from loaded geometry which will result in higher strength values. The variation of the tested sample is shown in Figure S3. The variations are due to the non-uniform surface roughness, microstructures and compositions at tested locations. However, it is clear in that the yield strength and peak strength of the sample after LSP plus LS are much higher than those after LS, and as received conditions.

**Supplementary Figure S3 │**Variation of stress strain curves under three conditions: (a) As received; (b) laser sintering of 2 wt. % of MWNTs; (c) Laser shock peening plus laser sintering of 2 wt. % MWNTs.

**REFERENCES**

1. Stuart SJ, Tutein AB, Harrison JA. A reactive potential for hydrocarbons with intermolecular interactions. *The Journal of Chemical Physics* **112**, 6472-6486 (2000).

2. Foiles SM, Baskes MI, Daw MS. Embedded-atom-method functions for the fcc metals Cu, Ag, Au, Ni, Pd, Pt, and their alloys. *Phys Rev B* **33**, 7983-7991 (1986).

3. Mendelev MI, Han S, Srolovitz DJ, Ackland GJ, Sun DY, Asta M. Development of new interatomic potentials appropriate for crystalline and liquid iron. *Philosophical Magazine* **83**, 3977-3994 (2003).

4. Eshelby JD. The Determination of the Elastic Field of an Ellipsoidal Inclusion, and Related Problems. *Proc R Soc Lon Ser-A* **241**, 376-396 (1957).

5. Böyükata M, Borges E, Braga JP, Belchior JC. Size evolution of structures and energetics of iron clusters: Molecular dynamics studies using a Lennard–Jones type potential. *Journal of Alloys and Compounds* **403**, 349-356 (2005).

6. Hashemi Haeri H, Ketabi S, Hashemianzadeh S. The solvation study of carbon, silicon and their mixed nanotubes in water solution. *J Mol Model* **18**, 3379-3388 (2012).

7. Alexander S. Visualization and analysis of atomistic simulation data with OVITO–the Open Visualization Tool. *Modelling and Simulation in Materials Science and Engineering* **18**, 015012 (2010).

8. Fabbro R, Fournier J, Ballard P, Devaux D, Virmont J. Physical study of laser‐produced plasma in confined geometry. *Journal of Applied Physics* **68**, 775-784 (1990).

9. Jeffrey W. Gilman, David L. VanderHart, and Takashi Kashiwagi, Thermal Decomposition Chemistry of Poly(vinyl alcohol), *Fire and Polymers II.* **July 21, 1995**, 161-185. Doi:10.1021/bk-1995-0599.ch011

10. Peng, Zheng, and Ling Xue Kong. "A thermal degradation mechanism of polyvinyl alcohol/silica nanocomposites." *Polymer degradation and stability*92.6 (2007): 1061-1071.
